# Shape-Memory Terpolymer Rods with 17-β-estradiol for the Treatment of Neurodegenerative Diseases: an ***In Vitro*** and ***In Vivo*** Study

**DOI:** 10.1007/s11095-016-2019-9

**Published:** 2016-09-14

**Authors:** Artur Turek, Edyta Olakowska, Aleksandra Borecka, Henryk Janeczek, Michał Sobota, Joanna Jaworska, Bożena Kaczmarczyk, Bożena Jarząbek, Arkadiusz Gruchlik, Marcin Libera, Arkadiusz Liśkiewicz, Halina Jędrzejowska-Szypułka, Janusz Kasperczyk

**Affiliations:** 1 Centre of Polymer and Carbon Materials, Polish Academy of Sciences, M. Curie-Sklodowskiej 34, Zabrze, Poland; 2 School of Pharmacy with the Division of Laboratory Medicine in Sosnowiec, Medical University of Silesia, Katowice, Chair and Department of Biopharmacy, Jednosci 8, Sosnowiec, Poland; 3 School of Medicine in Katowice, Medical University of Silesia, Katowice, Department of Physiology, Medykow 18, Katowice, Poland

**Keywords:** 17-β-estradiol, degradation, poly(L-lactide-co-glycolide-trimethylenecarbonate), rods, zero-order release

## Abstract

**Purpose:**

Estradiol (E2)-loaded poly(L-lactide-co-glycolide-trimethylenecarbonate) (P(L-LA:GA:TMC)) rods with shape-memory were developed for the treatment of neurodegenerative diseases. Usefulness of the extrusion method in the obtaining process was also considered. The influence of structural and surface properties during hydrolytic degradation was developed. The possible therapeutic aspect of rods with E2 was determined.

**Methods:**

The extruded rods were incubated in a PBS solution (pH 7.4, 37°C, 240 rpm). The amount of released E2 *in vitro* conditions was estimated by UV-VIS method. The following methods in the degradation of rods were applied: NMR, DSC, FTIR, GPC, SEM, and optical microscopy. Changes in water uptake and weight loss were also determined. *In vivo* study was performed on rats. Measurements of E2 level were performed before and after ovariectomy of rats using ELISA method. A sample of tissue adjacent to the site of the rod implantation was analysed under an optical microscope.

**Results:**

A stable and steady degradation process ensured zero-order release of E2. The *in vivo* study indicated a significant increase in the E2 level in serum after ovariectomy. Moreover, structural and surface features indicated that the extrusion method was appropriate for obtaining E2-loaded rods.

**Conclusions:**

Shape-memory P(L-LA:GA:TMC) rods with E2 are an adequate proposal for further research in the field of neurological disorders.

## Introduction

17-β-estradiol (E2) is an important hormone that regulates many functions in the body. This hormone’s reproductive role has extensively been described in the past few years. Individual therapeutic problems require various ways, periods and places of administration. For this reason, E2 is available in various pharmaceutical formulations, e.g. as oral tablets, vaginal tablets, or as transdermal therapeutic systems (1,2). However, recent studies have suggested that E2 may exhibit neuroprotective and antiapoptotic activity in the nervous system. Animal studies have shown that chronic E2 depletion increased injury size in the brain. Exogenous administration of E2 reduced infarct size following cerebral ischemia in cases of ovariectomized female rats and mice. Likewise, female rodents had greater survival rates, smaller infarct volume and lower cell loss as compared to males (3–5). Moreover, it was shown that the E2 level is related to both incidence and progression of various neurological disorders in humans and plays especially a protective role in neurodegenerative diseases (6). In the future, E2 replacement therapy may be administered in various neurodegenerative disorders in order to diminish the extent of disease-related brain damage or to design effective neuroprotective agents that can be administered in clinical practice. Hence, novel possibilities in E2 therapy should influence the development of drug delivery systems. The therapy of neurological disorders requires sustained release of the drug substance, therefore known “classical” drug formulations with E2 may be insufficient for this purpose. To this moment, developments in drug formulation technology to block estrogens, hormonal contraception and hormone replacement therapy have been observed (7), e.g. implant rods, microspheres, vaginal ring, thermosensitive gel, hormone-DNA complexes, and a delivery system based on cationic liposomes (8–11).

For this purpose, aliphatic polyesters have been recommended most often, i.e. polylactic acid (12,13), poly(lactide-co-glycolide) (PLGA) (14–16), poly-ε-caprolactone (12), as well as poly-ε-caprolactone/ PLGA blends (8).

The preferred features for sustained release of E2 are safety, low invasiveness and regular release of the drug substance. In this work, implantable rods were developed based on an innovative bioresorbable terpolymer poly(L-lactide-co-glycolide-trimethylenecarbonate) P(L-LA:GA:TMC) with shape-memory (17,18) and synthesized with the use of a low-toxic initiator of polymerization, i.e. zirconium (IV) acetylacetonate (Zr(Acac)_4_) (19).

The use of an initiator exhibiting low toxicity seems to be particularly important in the case of drug carriers for prolonged release – with regard to its possible interactions with tissues. Commercially available polyesters and polyestercarbonates are commonly synthesized using stannous compounds as initiators. However, they are relatively toxic (20). Zr(Acac)_4_ seems to be a promising alternative, and its low biological toxicity as compared to stannous octanoate has been confirmed (21). Copolymers and terpolymers obtained with this initiator were characterized in detail in a previous study (18,22). Moreover, the Zr(Acac)_4_ initiator makes it possible to achieve a similar chain microstructure of polymeric materials based on lactidyl, glicolidyl, carbonate and caproyl monomers such as stannous octanoate and allows to obtain high-molecular-weight copolymers with good mechanical properties (23,24).

The biodegradable shape-memory terpolymer proposed in this study may reduce invasiveness and influence regular release. Generally, shape-memory polymers are the most promising solution in biomedical applications (25,26). They have found application mainly in the production of drug-eluting stents. They are used in the treatment of arterial stenosis and in the prevention of restenosis. For this purpose, acetylsalicylic acid, dexamethasone, and mitomycin C with curcumin were proposed (27,28). In stents, shape-memory polymers play a double role, i.e. in the maintenance of tissues and in drug delivery. However, other solutions besides stents are also possible. In this work, a thermally sensitive P(L-LA:GA:TMC) terpolymer was used which is able to move from a temporary fixed shape to the original permanent shape upon exposure to a thermal stimulus (18). Therefore, optimization of treatment of neurodegradative diseases by reducing invasiveness and the burst effect may be guaranteed. The burst effect is a phenomenon that is caused by solvent penetration of the surface. Our concept assumes a reduction in the surface size and thus a reduction of the burst effect *ipso facto*. The proposed rods will possess a smaller diameter and greater length before implantation as well as a greater diameter and smaller length after implantation, therefore reduction of both the surface size and invasiveness will be possible. Additionally, biodegradability allows to avoid explantation.

The aim of this work was to study P(L-LA:GA:TMC) rods with shape-memory containing E2 for the treatment of neurodegenerative diseases. Usefulness of the extrusion method in the obtaining process was also considered on the basis of E2 and P(L-LA:GA:TMC) thermal properties. The study was performed *in vitro* and *in vivo*. In the former, the influence was tested of the rods’ structural and surface properties on E2 release during hydrolytic degradation. In the latter, the possible therapeutic aspect was determined of P(L-LA:GA:TMC) rods with E2.

## Materials and Methods

### Polymer

A shape-memory terpolymer P(L-LA:GA:TMC) with a ratio of 57:19:24 (59.0 kDa) and an average length of lactidyl (*l*_*LL*_), glycolidyl (*l*_*GG*_) and carbonate (*l*_*TMC*_) blocks as 4.1, 1.1 and 1.5, respectively, was synthesized with the use of a low-toxic initiator Zr(Acac)_4_ (Sigma-Aldrich) at the Centre of Polymer and Carbon Materials of the Polish Academy of Sciences in Zabrze according to a previously developed methodology (19).

### Extrusion Process

P(L-LA:GA:TMC) was used to prepare rods containing 10% w/w of E2 (Sigma, USA) by the hot melt extrusion method. Before the process, raw terpolymer was dried on air and subjected to grinding at a temperature of −196°C in a cryogenic mill (6870 SPEX, USA). Then E2 was introduced to the milled terpolymeric material. The mixture was vortexed and subsequently placed for 14 days in a vacuum oven with a temperature of 23°C and a pressure of 80 mbar. The mixture of P(L-LA:GA:TMC) and E2 was fed to an extruder cylinder heated to 105°C. This process was carried out in a co-rotating twin screw extruder (Minilab, Thermo-Haake, GE) using a plasticizing screw rotational speed of 20 rpm.

For the *in vitro* model, the molten mixture was extruded through a 0.7 mm diameter die. The molded rod was received on a chilled roll. Afterwards, rods 1 mm in diameter and 10 mm in length were formulated. For the *in vivo* model the mixture was extruded through a 1.0 mm diameter die in the same conditions as previously. The final product was a rod 1.5 mm in diameter and 10 mm in length.

### *In Vitro* Study

#### The *In Vitro* Degradation Process of Terpolymer Rods

The rods were placed in a PBS (phosphate buffered saline) solution (pH 7.4) (Sigma-Aldrich) and incubated under constant conditions at a temperature of 37°C and shaking at 240 rpm during 113 days.

#### E2 Release in the *In Vitro* Study

The amount of released E2 was estimated by the UV-VIS method. The supernatants were freeze-dried and the dry mass of samples was dissolved in 1 ml of methanol. Optical absorption measurements of the solutions were performed at room temperature on a V-570 double-beam UV-Visible/NIR spectrometer (Jasco Analytical Instruments, USA). A deuterium lamp was used as a source of ultraviolet and a halogen lamp was used for the visible and near-infrared parts of the light spectrum.

Absorption spectra of E2 dissolved in pure methanol were recorded with the use of quartz cells within a spectral range from 200 to 230 nm, where the absorption bands were observed. The main absorption band of E2 was at 203.5 nm; a calibration curve was prepared for this band and the calibration equation was estimated.

#### Polymer Composition and Chain Microstructure Study

Both the composition and chain microstructure study of P(L-LA:GA:TMC) samples were determined by nuclear magnetic resonance spectroscopy (NMR). Spectra were recorded using a Bruker-Avance II Ultrashield Plus spectrometer operating at 600 MHz (^1^H) and 150 MHz (^13^C) using DMSO-d6 as a solvent, with a 5-mm sample tube. ^1^H NMR spectra were obtained with 32 scans, 11 μs pulse width and 2.65 s acquisition time, ^13^C NMR spectra were obtained with 20,000 scans, 9.4 μs pulse width and 0.9 s acquisition time. Signals observed in ^1^H and ^13^C NMR spectra were assigned to the appropriate sequences in the polymer chain according to a previously described procedure (29). The content of the monomer units of lactide (*F*_*LL*_), glycolide (*F*_*GG*_), and carbonate (*F*_*TMC*_) as well as *l*_*LL*_, *l*_*GG*_ and *l*_*TMC*_ in the terpolymer chain were calculated.

#### Thermal Study

Thermal analysis of P(L-LA:GA:TMC) and E2 samples was carried out by means of the differential scanning calorimetry (DSC) method. The TA DSC 2010 apparatus (TA Instruments, New Castle, DE) was used during the measurement. The instrument was calibrated with high-purity indium and gallium and worked under a nitrogen atmosphere (flow rate 50 ml/min).

The terpolymer material was heated at a rate of 20°C/min. During the first run the rod samples were heated to 200°C, then the melted samples were rapidly cooled to −20°C. At the second run, the rods were heated within a range of −20 to 200°C. The glass transition temperature (*T*_*g*_) was determined as the midpoint of the heat capacity change of the amorphous sample, from the second heating run.

However, the DSC measurements for E2 were performed as follows: (i) first heating run at 20°C/min, (ii) cooling run at 20°C/min, (iii) second heating run after cooling at 20°C/min, and (iv) second heating run after quenching.

#### E2 - P(L-LA:GA:TMC) Interaction Study

The transmission technique of Fourier transform infrared spectroscopy (FTIR) was used to determine the E2 - P(L-LA:GA:TMC) interaction. Infrared spectra were recorded on a DIGILAB FTS-40A Fourier transform infrared spectrometer (Bio-Rad, USA) in the range of 4000–400 cm^−1^ at a resolution of 2 cm^−1^ and for an accumulated 32 scans.

The E2 sample was analyzed in the form of pellets in potassium bromide, the other compounds were analyzed as films after dissolving in acetone and evaporating onto potassium bromide windows.

#### Molecular Weight and Molecular Weight Distribution Study

Molecular weight (*M*_*n*_) and molecular weight distribution (*D*) of the samples were determined by gel permeation chromatography (GPC) using a Viscotek Rimax chromatograph with two Viscotek 3580 columns and a Shodex SE 61 detector. The process was carried out with chloroform as a solvent with a flow rate of 1 ml/min. The molecular weights were calibrated with polystyrene standards.

#### Water Uptake and Weight Loss Study

Changes in the water uptake (*WU*) and weight loss (*WL*) for P(L-LA:GA:TMC) rods during the degradation process were calculated according to the following equations:

1 $$ WU\left[\%\right]=\left[\left({M_{we}}_{\mathrm{t}}-{M}_{dry}\right)/{M_{we}}_{\mathrm{t}}\right)\times 100 $$

2 $$ WL\left[\%\right]=\left[\left({M}_0-{M}_{dry}\right)/{M}_0\right)\times 100 $$

where:

*WU*: water uptake
*WL*: weight loss
*M*_*wet*_: wet mass of rod
*M*_*dry*_: dry mass of rod
*M*_*0*_: initial mass of rod.

#### Morphology Study of Drug Formulation

Both the morphology of the surface and cross-section of the P(L-LA:GA:TMC) rod with E2 were characterized by optical and scanning electron microscopy (SEM).

The optical microscope Carl Zeiss (Opton-Axioplan, PL) was used for structure identification of the surface and cross-section of the P(L-LA:GA:TMC) rod with E2 at a magnification of ×200.

Electron micrographs were obtained using a scanning electron microscope (Quanta 250 FEG, FEI Company, USA) operating with an acceleration voltage of 5 kV under low vacuum conditions (80 Pa) from secondary electrons collected by a Large Field Detector. The samples were mounted on microscope stubs with the use of double-sided adhesive carbon tape.

### *In Vivo* Study

#### Sterilization Process of the Rods

Sterilization of the rods was performed using an electron beam accelerator (10 MeV, 360 mA). The absorbed dose was 25 kGy. Sterilization was conducted at the Institute of Nuclear Chemistry and Technology of the Center for Radiation Research and Technology (Certificate No. 457/2014/E).

#### Animal Model

The experiment protocol was approved by the Local Ethics Committee for Animal Experimentation of the Medical University of Silesia, Katowice (Permission No. 10/2015). Fourteen adult female Wistar rats (weighing 250–300 g) were obtained from the Center for Experimental Medicine of the Medical University of Silesia, Katowice. The rats were randomly divided into 3 groups, i.e. (i) control (*n* = 6), (ii) ovariectomized group and treated with rods with E2 in a dose of 2.5 mg (*n* = 4), (iii) ovariectomized group and treated with rods with E2 in a dose of 5 mg (*n* = 4).

The animal study was performed according to accepted standards of animal care (i.e. National Institute of Health, Guide for Care and Use of Laboratory Animals).

After the animals were anesthetized (i.e. ketamine 100 mg/kg and xylazine 10 mg/kg b.w.), dorsolateral incisions were made on the back, both the right and left horns of the uterus were exposed and the ovaries were carefully removed, leaving the uterus intact.

Seven days after the ovariectomy in group 2 and group 3 of the animals, sterilized rods (25 kGy) with E2 in doses of 2.5 and 5 mg, respectively, were implanted subcutaneously after an incision in the back of the neck. Then the skin was sutured with one stitch. In the control group the animals were subjected only to an ovariectomy without implantation of E2 rods and without surgical intervention.

The animals were anesthetized and blood was collected from the ophthalmic artery for E2 concentration measurements.

#### Determination of the E2 Serum Level

Measurements of the E2 level were performed before the ovariectomy and 7, 14, 21 and 28 days after the ovariectomy procedure. The E2 concentration in the serum was measured using an ELISA KIT (Estradiol ELISA KIT, Demeditec Diagnostics GMBH, Kiel, GE). All animals were deeply anesthetized and decapitated after 4 weeks.

Prism 5.01 software (GraphPad Software, San Diego, CA, USA) was used for the statistical analysis. Data were presented as mean ± S.E.M. Analyses of the E2 concentration in serum were performed using two-way ANOVA with the Bonferroni *post hoc* test. Values of *p* < 0.05 were considered to be statistically significant.

#### Morphology Study of Rat Tissue

A sample of tissue adjacent to the site of the rod implantation was removed and immediately fixed in 10% formalin solution. After 24 h the sample was immersed in 15% saccharose solution for 6 h, and for the next 12 h in 30% saccharose solution. After this time period the sample was fixed in special tissue embedding medium (Thermo Scientific, USA) and was cut with the use of a microtome into 10-micrometer-thick sections and placed on glass slides.

All sections were fixed in xylene for 15 min and then in 96% ethanol for 15 min. After rinsing with water the sections were stained with hematoxylin for 20 min and with eosin for 5 min. Then all sections were rinsed with water, 96% ethanol, xylene, dried and analyzed under a microscope at a magnification of ×40 (Ecotone EV-157, Poland).

## Results

### Thermal Characterization of E2 and Raw P(L-LA:GA:TMC)

**Fig. 1:**
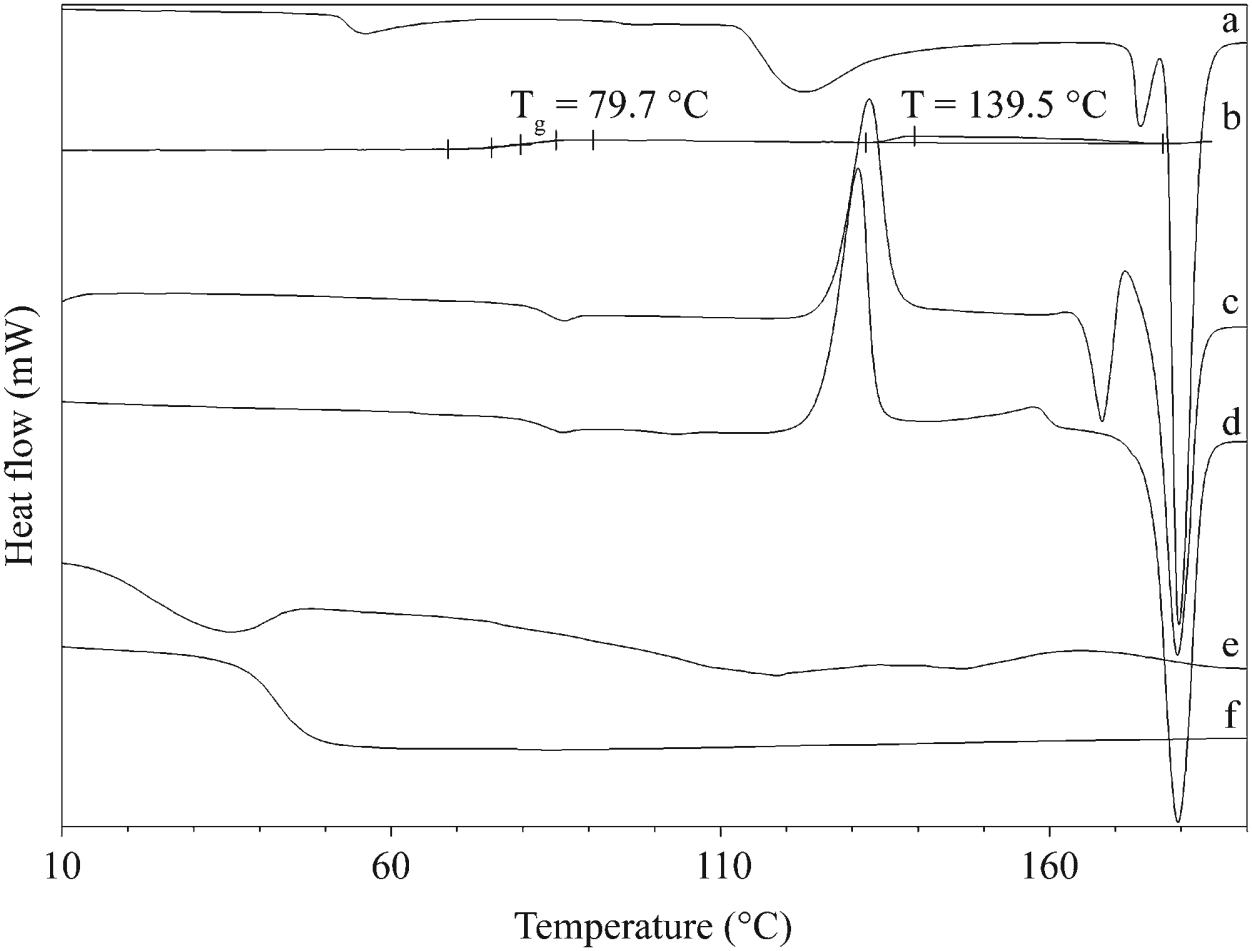
DSC curves of the first heating run for E2 (**a**), cooling run for E2 (**b**), second heating run after cooling at 20°C/min for E2 (**c**), second heating run after quenching for E2 (**d**), first heating run for P(L-LA:GA:TMC) (**e**) and second heating run after cooling at 20°C/min for P(L-LA:GA:TMC) (**f**).

DSC study of E2 and raw P(L-LA:GA:TMC) was performed to define thermal conditions for extrusion process. For E2, three endothermic peaks with maxima at a temperature of 122.4°C (*ΔH* = 55.5 J/g), 173.8°C (*ΔH* = 9.3 J/g), and 179.7°C (*ΔH* = 107.5 J/g) were observed in the first heating run at 20°C/min (20–200°C) (Fig. 1a). The first two endotherms correspond to the partial release of hydrogen-bonded water and the complete loss of lattice water. The third endotherm reflected melting, thus indicating a crystalline character. Cooling at 20°C/min of the E2 sample from the melt revealed the presence of *T*_*g*_ at 79.7°C and a smallbroad exotherm with a maximum at 139.5°C (Fig. 1b).

The second heating runs were performed for samples cooled in various ways, i.e. by cooling at 20°C/min and by quenching (Fig. 1c, Fig. 1d). In the first case, *T*_*g*_ was observed at 82.2°C with the presence of one exothermic peak at 132.6°C, and the next two endothermic peaks were noted at 167.9°C and 179.4°C. In the second case, *T*_*g*_ was observed at the same value followed by double crystallization exothermic peaks (130.9°C and 157.8°C) and one endothermic peak at 179.5°C.

The thermal analysis of raw powder of P(L-LA:GA:TMC) revealed no significant endothermal and exothermal events during the first heating run (Fig. 1e). *T*_*g*_ determined during the second heating run at 20°C/min was 42.6°C after previous cooling at 20°C/min (Fig. 1f).

### E2 - P(L-LA:GA:TMC) Interaction Study

**Fig. 2:**
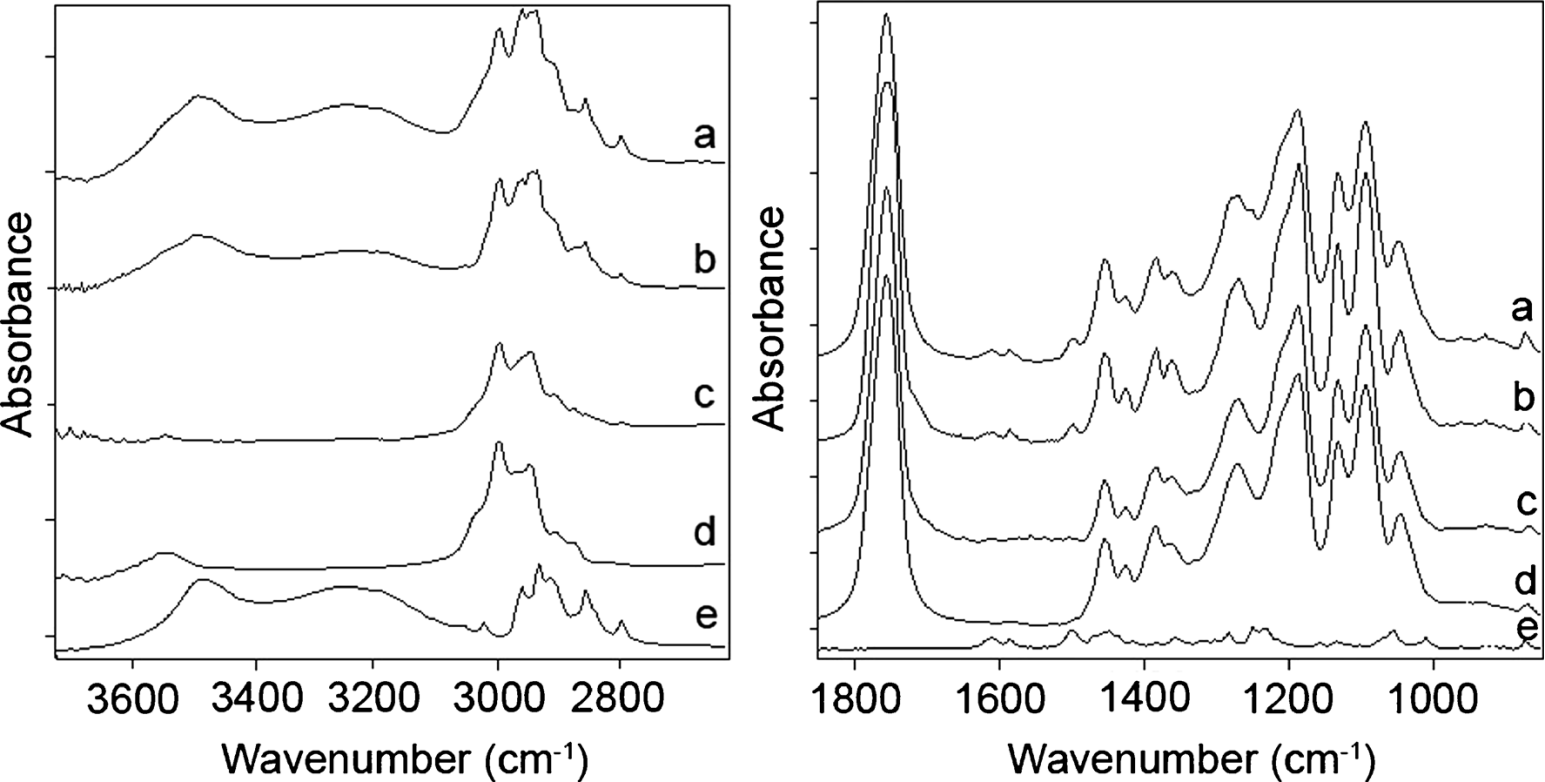
Infrared spectra of the sum of raw powder of P(L-LA:GA:TMC) and E2 spectra (**a**), mixture of P(L-LA:GA:TMC) and E2 (**b**), P(L-LA:GA:TMC) rod with E2 (**c**), raw powder of P(L-LA:GA:TMC) (**d**) and E2 (**e**). No differences between spectrum a and spectrum b indicate a lack of E2- P(L-LA:GA:TMC) interactions.

The FTIR spectrum of E2 revealed two bands in the region of 3600–3100 cm^−1^, i.e. the first band at 3445 cm^−1^ and the second, broader band at 3230 cm^−1^ (Fig. 2a). These bands correspond to the stretching vibrations of the hydroxyl group and derive from OH groups of E2 molecules as well as from hydrogen-bonded and lattice water.

For P(L-LA:GA:TMC), a band at 3503 cm^−1^ was observed, which may be interpreted as an overtone of the strongest intensity band at 1757 cm^−1^ resulting from the stretching vibrations of the C=O ester group (Fig. 2b).

Both for E2 and P(L-LA:GA:TMC), bands in the region of 3000–2800 cm^−1^ (stretching mode), 1480–1350 cm^−1^ and 1100–800 cm^−1^ (deformations) and attributed to the vibrations of the CH_2_ and CH group were observed.

Bands characteristic of the stretching vibrations of the C-O-C and C-OH groups appeared in the region of 1250–1050 cm^−1^. An aromatic ring present in the E2 compound was absorbed in the region of 3100–3000 cm^−1^ (stretching of C-H), 1610–1480 cm^−1^ (stretching of phenyl ring), 1100–800 cm^−1^ (C-H deformations).

In the spectrum of the P(L-LA:GA:TMC) rod with E2, bands characteristic of E2 were not observed due to its low content. For that reason, the spectrum of the mixture of P(L-LA:GA:TMC) and E2 in a ratio of 1:1 was recorded.

### Morphology Study of Drug Formulation

**Fig. 3:**
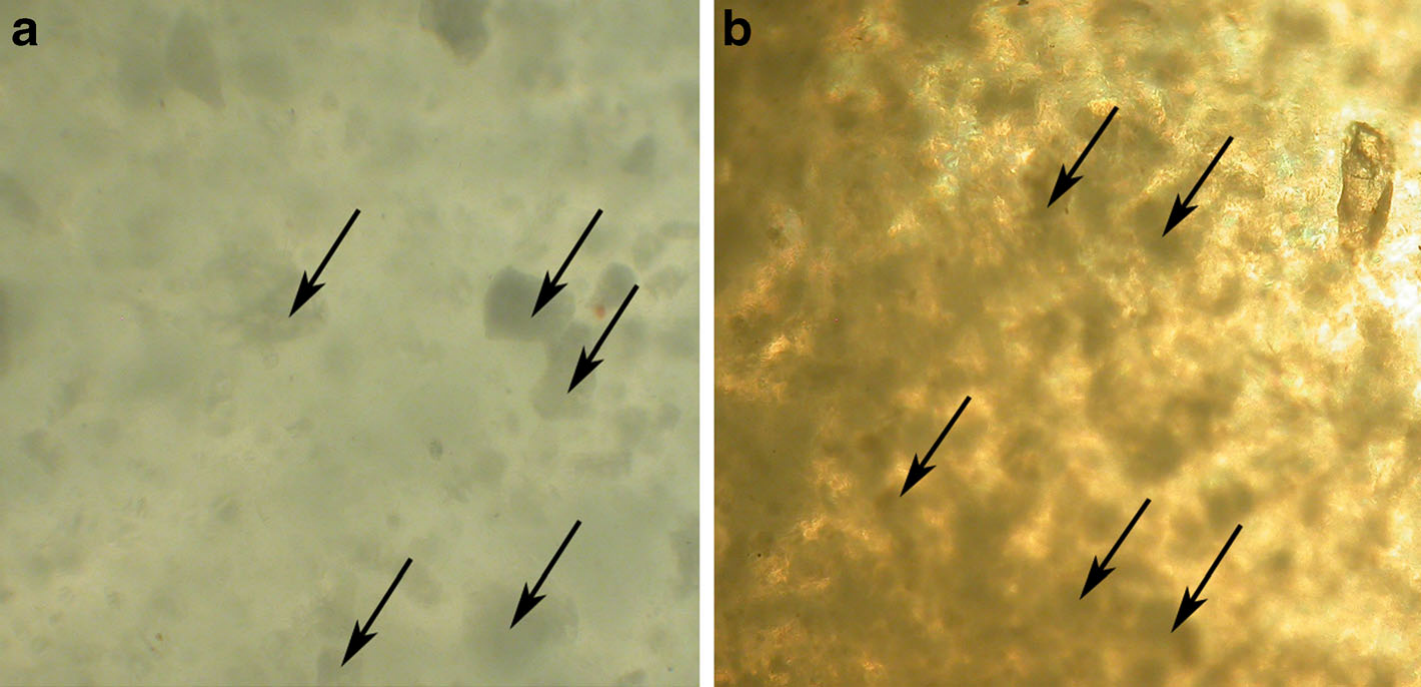
Optical microscopy images of the cross-section (**a**) and surface (**b**) of P(L-LA:GA:TMC) with E2 (magnification × 200).

Observation of the P(L-LA:GA:TMC) rod with E2 performed by optical microscopy revealed a non-homogeneous character. Particles of various size were visible in the cross-section (Fig. 3a) and on the surface (Fig. 3b). Some examples of particles were marked in figures with arrows.

### *In Vitro* Study

#### E2 Release from P(L-LA:GA:TMC) Rods

**Fig. 4:**
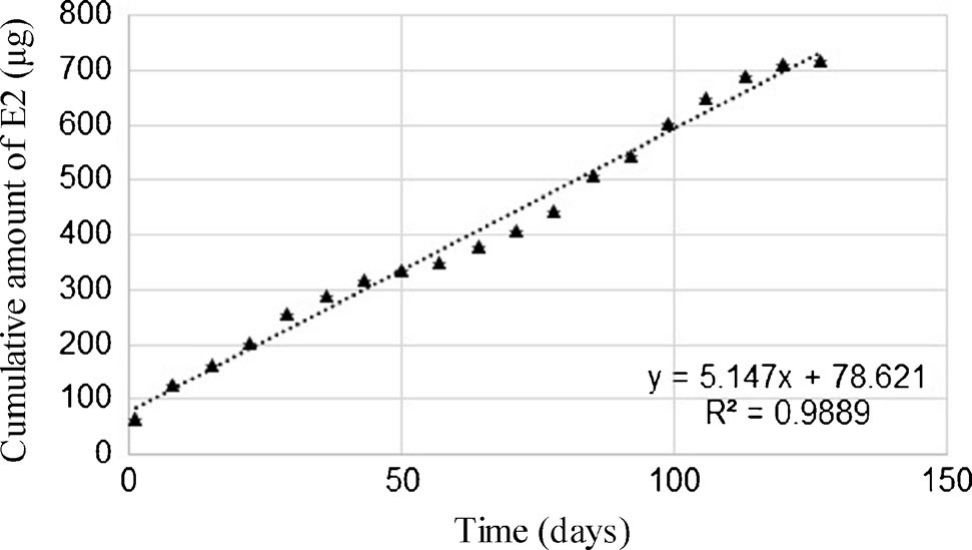
*In vitro* cumulative release profile of E2 from P(L-LA:GA:TMC) rods (*n* = 10) fitted in the zero-order release.

A total of 718.45 ± 4.85 μg (*n* = 10) of E2, i.e. 100% of the introduced substance, was released from the P(L-LA:GLA:TMC) rods during a period of 113 days. A zero-order kinetic model was used to describe the release of E2 (Fig. 4).

3 $$ {\mathrm{Q}}_{\mathrm{t}}={\mathrm{Q}}_0+{\mathrm{k}}_0\mathrm{t}, $$

where

Q_t_: cumulative amount in μg of E2 released in time t
Q_0_: initial amount in μg of E2 released in time t = 0
k_0_: zero-order release constant (release rate) (μg/day).

The plot of Q_t_ versus t is a straight line with a slope of k_0_. The straight line of linear regression analysis indicates a zero-order release kinetic of E2 with an R^2^ value of 0.9889. The zero-order release constant k_0_ was 5.15 μg per day. The initial amount of E2 released after the first day (for t = 0) was at a level of 8.9 ± 0.0016%.

#### Polymer Composition and Chain Microstructure Changes

**Fig. 5:**
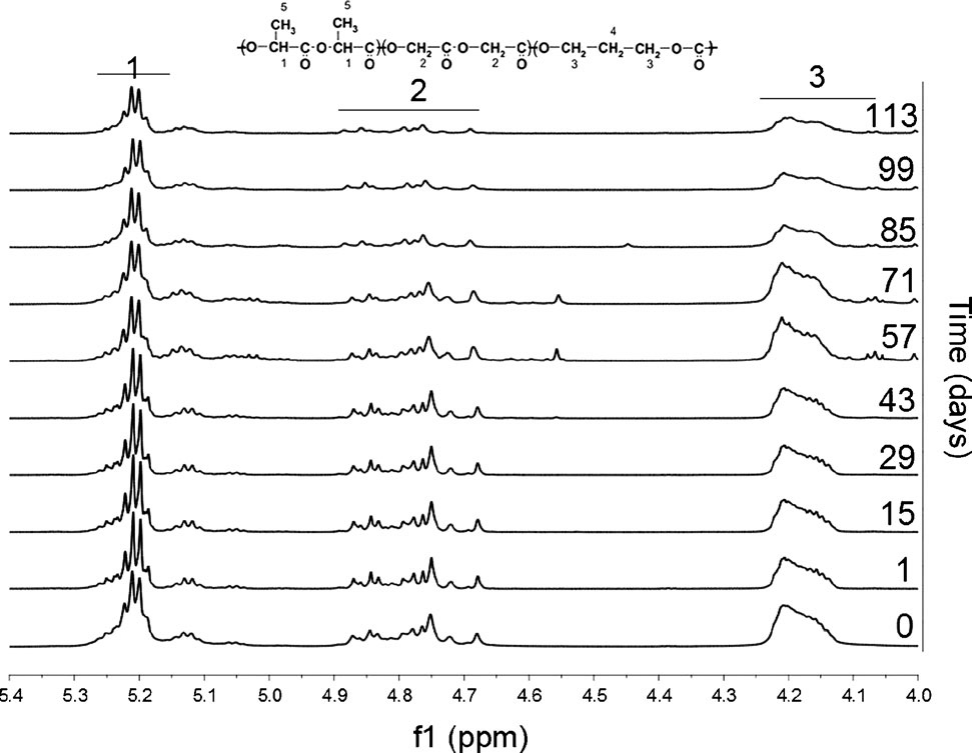
H^1^ NMR spectra (600 MHz, DMSO-d6) of the P(L-LA:GA:TMC) rod with E2 during 113 days of degradation (methine proton region of the lactidyl units (*1*) and methylene proton region of the glycolidyl (*2*) and carbonate units (*3*)).

Changes in the monomer unit distribution in the polymer chain of P(L-LA:GA:TMC) during the degradation process were determined on the basis of ^1^H NMR spectra (Fig. 5). P(L-LA:GA:TMC) initially contained a predominant *F*_*LL*_ (above 58 mol%) as well as a low of the *F*_*GG*_ (~18 mol%) and *F*_*TMC*_ units (~24 mol%) (Table I).

**Table I:** Parameters Characterizing E2-Loaded Rods During 113 days of Incubation

Time (days)	*F*_*LL*_ (mol%)	*F*_*GG*_ (mol%)	*F*_*TMC*_ (mol%)	*l*_*LL*_	*l*_*GG*_	*l*_*TMC*_	1st run				2nd run	*M*_*n*_ (kDa)	*D*	*WU* (%)	*WL* (%)
							*T*_*m1*_ (°C)	*∆H*_*1*_ (J/g)	*T*_*m2*_ (°C)	*∆H*_*2*_ (J/g)	*T*_*g*_ (°C)				
0	57.9	17.6	24.5	4.2	1.1	1.5	*ND*	*ND*	126.7	21.3	40.2	45.8	1.9	0	0
1	57.3	17.7	25.0	4.1	1.1	1.6	*ND*	*ND*	139.4	5.1	39.4	43.1	1.9	0.9	0
15	57.1	17.6	25.3	3.8	1.1	1.5	*ND*	*ND*	132.0	13.3	38.7	33.4	1.9	4.0	0.5
29	57.5	17.5	25.0	3.8	1.1	1.5	86.6	1.4	141.1	7.9	38.2	18.8	1.9	6.8	2.2
43	57.4	17.2	25.3	3.2	1.2	1.5	81.4	4.8	136.4	9.9	35.7	8.8	2.3	8.7	3.4
57	57.1	14.7	28.2	3.1	1.2	1.6	71.1	4.1	116.6	1.5	22.6	1.4	2.5	12.9	4.9
71	58.2	13.2	28.7	2.5	1.1	1.6	75.8	8.5	137.2	16.1	25.3	1.3	1.9	34.3	31.0
85	62.7	12.3	25.0	*ND*	*ND*	*ND*	85.4	13.9	139.7	21.3	34.9	1.2	1.9	38.9	38.3
99	64.2	11.9	23.9	*ND*	*ND*	*ND*	88.6	17.5	145.8	25.6	38.7	1.1	1.9	66.1	68.8
113	64.0	11.5	24.5	*ND*	*ND*	*ND*	94.9	18.9	151.3	29.9	41.3	0.8	3.5	76.5	86.7

*F*_*LL*_ molar percentage of lactidyl units, *F*_*GG*_ molar percentage of glycolidyl units, *F*_*TMC*_ molar percentage of carbonate units, *l*_*LL*_ average length of lactidyl blocks, *l*_*GG*_ average length of glycolidyl blocks, *l*_*TMC*_ average length of carbonate blocks, *T*_*m1*_ and *T*_*m2*_ melting temperatures, *∆H*_*m1*_ *and ∆H*_*m2*_ enthalpies of melting, *T*_*g*_ glass transition temperature, *M*_*n*_ molecular weight, *D* molecular weight distribution, *WL* weight loss, *WU* water uptake, *ND* non-detected

The NMR study revealed differences in monomer unit distribution during the 113-day period of incubation. Enhanced degradation of the glycolidyl units was noted: *F*_*GG*_ decreased from 17.6 mol% at the beginning of the experiment to 11.5 mol% at the last measured stage of the degradation process. In the same period, *F*_*LL*_ increased and *F*_*TMC*_ remained at the same level (Table I).

**Fig. 6:**
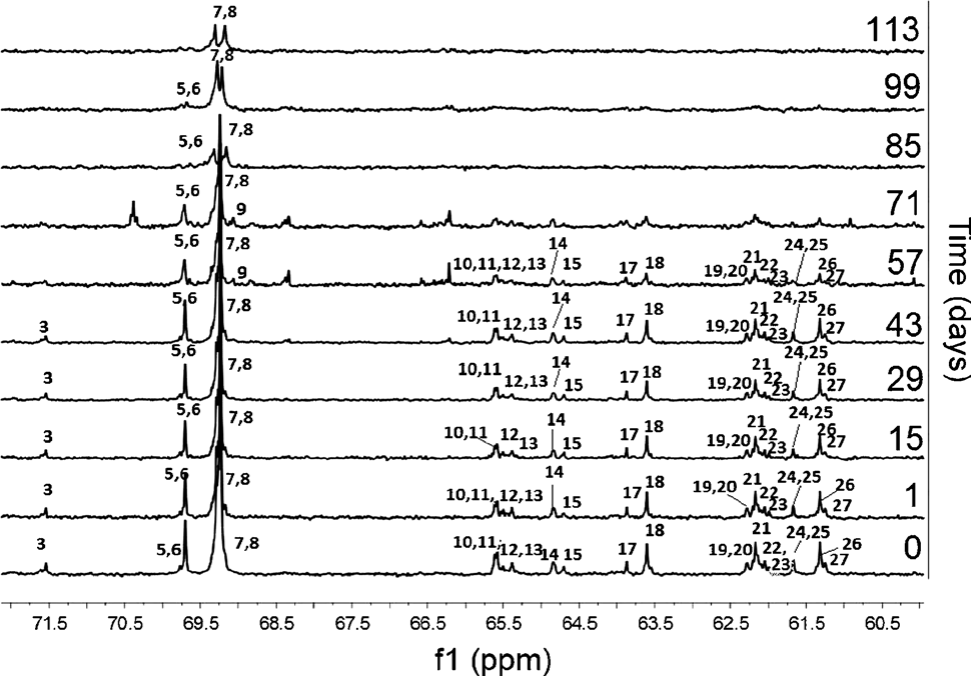
^13^C NMR (150 MHz, DMSO-d6) of the P(L-LA:GA:TMC) rod with E2 during 113 days of degradation (methine carbon region of the lactidyl units (*LL*) and methylene carbon region of the glycolidyl (*GG*) and carbonate units (*T*)). *3-TLLLT, 5-GLLT+GLLLT, 6-TLLLL+LLLLT, 7-LLLL, 8-LLGG, 9-GLG, 10-TT’G+GGT’GG+TGT’GG, 11-GGTT, 12-GGT’GT+GT’GT, 13-GGT’GT+TT’L+LTT’, 14-TT’T+TTT’+TT”G, 15-TT’GG, 17-TGT+TGGL+TGGT, 18- LGGGT+TGGGT+GGGGT, 19-TT’L+LT’T, 20-LT’L+LT”L, 21-GGT’T, 22-GGT”GT+GGT”GG, 23-TGT”GG+TGT’GT, 24-TGGGG+TGG, 25-TGGT, 26-GGLL, 27-GGGG.*

On the basis of P(L-LA:GA:TMC) ^13^C NMR spectra, changes in the chain microstructure were analyzed and described (Fig. 6). The analysis revealed a meaningful, steady decrease of *l*_*LL*_ during degradation. In the case of the *l*_*GG*_ and *l*_*TMC*_, no changes were noticeable during the whole process of degradation (Table I). After 70 days of the experiment, ^13^C NMR analysis was not possible due to an unsatisfactory resolution of the spectra connected with an advanced disintegration process of the terpolymer. Degradation of P(L-LA:GA:TMC) proceeded steadily.

#### Thermal Properties Study

DSC study of E2-loaded P(L-LA:GA:TMC) rods was performed to describe degradation process.

**Fig. 7:**
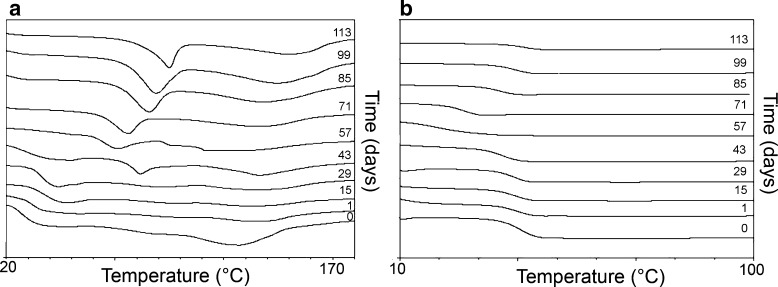
DSC curves of the first run (**a**) and second run (**b**) for E2-loaded P(L-LA:GA:TMC) rods after 0, 1, 15, 29, 43, 57, 71, 85, 99 and 113 days of degradation.

An analysis of the first run of rods revealed significant changes in the parameters, such as *T*_*m*_ and *∆H* during 113 days of degradation. In the period of 0 to 15 days, only one thermal event at DSC scans with high oscillation in the values of *T*_*m2*_ and *∆H*_*2*_ was observed (Fig. 7a). Additional endothermic peaks appeared (*T*_*m1*_) on the 29th day of the degradation process. In the period of the 29th to 57th day, a decrease in the *T*_*m1*_ *, T*_*m2*_ values was observed. Later, an increase up to day 113 was noted. It should be pointed out that in the same time period an increasing trend was observed for *∆H*_*1*_. In the case of *∆H*_*2*_, oscillation in the value was noted between the 29th and 57th day, however, from the 57th day onwards a phenomenon analogical to *∆H*_*1*_ was observed.

The second heating revealed noticeable changes in *T*_*g*_ (Fig. 7b). A significant decrease in *T*_*g*_, from 40.2 to 22.6°C, was noted after 57 days. After this time the value of *T*_*g*_ increased to 41.3°C.

#### Molecular Weight and Molecular Weight Distribution Study

**Fig. 8:**
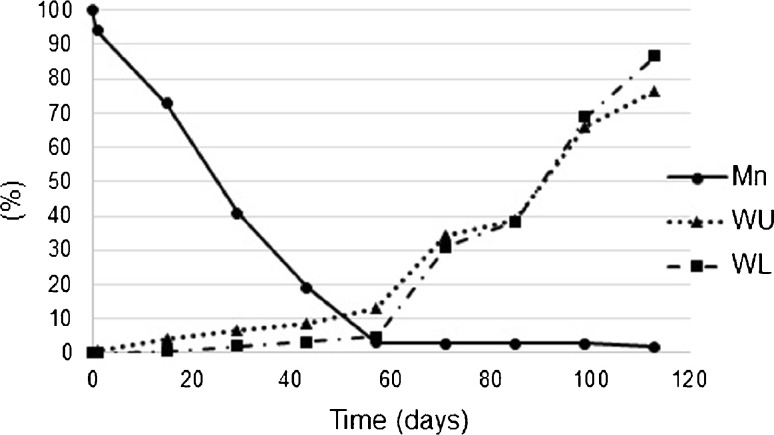
Comparison of *M*_*n*_ loss [%], *WU* [%] and *WA* [%] rise of E2-loaded P(L-LA:GA:TMC) rods after 0, 1, 15, 29, 43, 57, 71, 85, 99 and 113 days of degradation.

The GPC analysis revealed a gradual decrease of *M*_*n*_ during 113 days of degradation, from 45.8 to 0.8 kDa (Fig. 8). The most significant drop was observed in 43 and 57 day. Molecular weight distribution (*D*) was also determined. Dispersity increased from 1.9 to 2.5 after 57 days of incubation, then it decreased to 1.9 during the next 42 days. After this time *D* increased again to 3.5 at the end of the process.

#### Water Uptake and Weight Loss

Changes were noted in both the *WU* and *WL* values. An increasing trend for *WU* was observed during 113 days. During this period the amount of absorbed water was 76.5%. The weight dropped by 86.7% during the degradation process. The most significant changes in these parameters were observed after 57 day. Moreover, the correlation between these two parameters was 0.99 (Fig. 8).

#### SEM Study

**Fig. 9:**
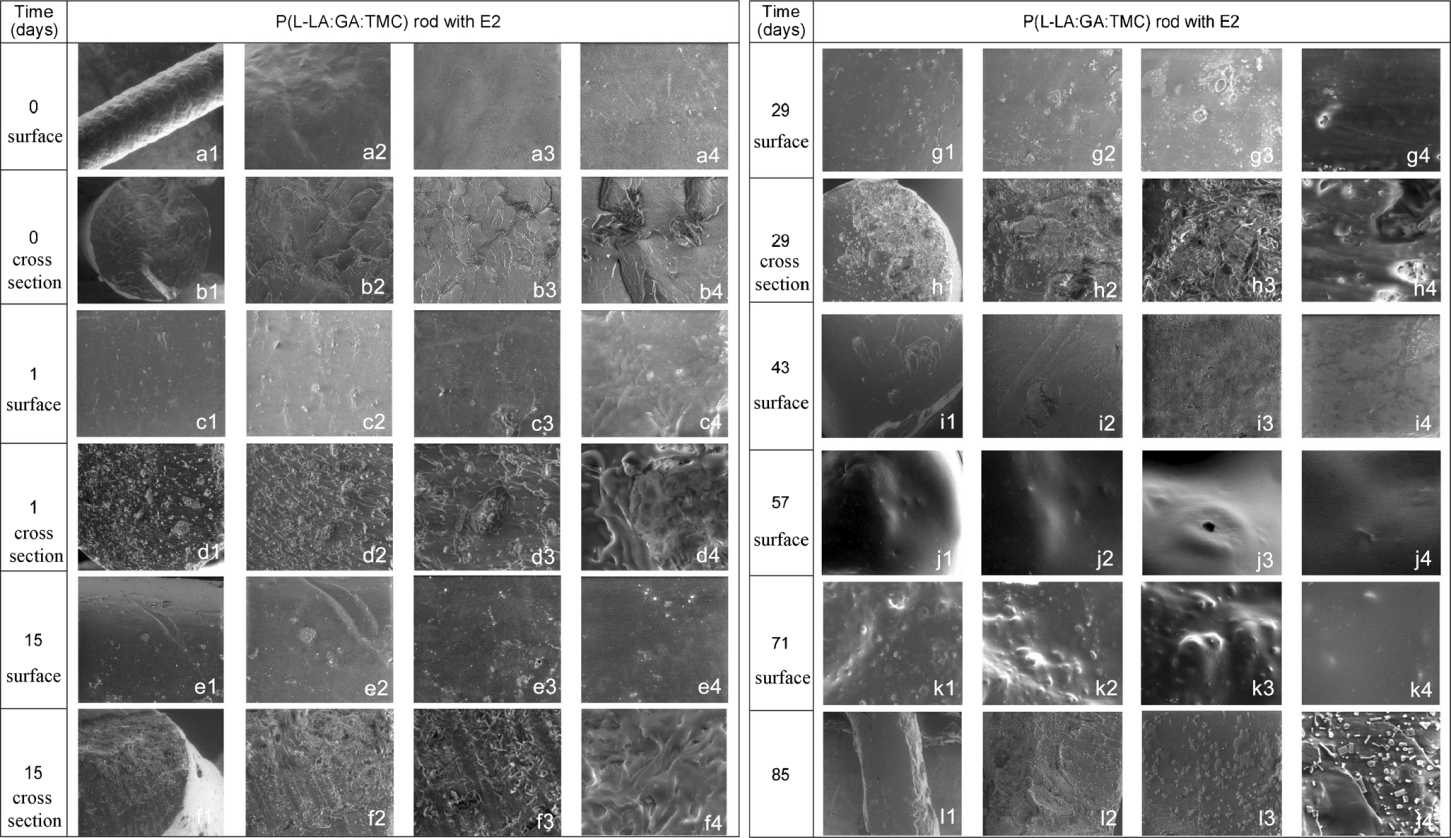
SEM images of P(L-LA:GA:TMC) rods with E2 degraded for 0 days (picture number-magnification) (a1-×115, a2-×423, a3-×2666, a4-×10436, b1-×238, b2-×1131, b3-×2468, b4-×9870), 1 day (c1-×500, c2-×1000, c3-×2500, c4-×10000, d1-×500, d2-×1000, d3-×2500, d4-×10000), 15 days (e1-×500, e2-×1000, e3-×2500, e4-×10000, f1-×500, f2-×1000, f3-×2500, f4-×5000, 29 days (g1-×500, g2-×1000, g3-×2500, g4-×10000, h1-×500, h2-×1000, h3-×2500, h4-×10000), 43 days (i1-×377, i2-×1000, i3-×2293, i4-×7500), 57 days (j1-×500, j2-×1000, j3-×2500, j4-×5000), 71 days (k1-×500, k2-×500, k3-×1000, k4-×5000), 85 days (l1-×183, l2-×755, l3-×2500, l4-×8409).

The outer morphology of native E2 rods performed by using the SEM method exhibited a solid surface. The inner morphology of native E2 rods also showed a solid structure, however, some layers and delamination as a result of preparing the cross-section were visible (Fig. 9).

SEM observations were performed for 85 days. The degradation indicated changes in both the outer and inner morphology of the rods. The surface started to differ in its morphological features, i.e. surface roughness and recesses were appeared. No evident porous surfaces, slits and cracks were observed on the surface during degradation (Fig. 9). However, the primary surface disappeared in 43 day.

The inner morphology revealed a solid structure from the beginning. Delamination was also visible as a result of preparing the cross-section. Because of rod features there was not possible to obtain cross-section from 43 day.

### *In Vivo* Study

#### Therapeutic Effect

**Fig. 10:**
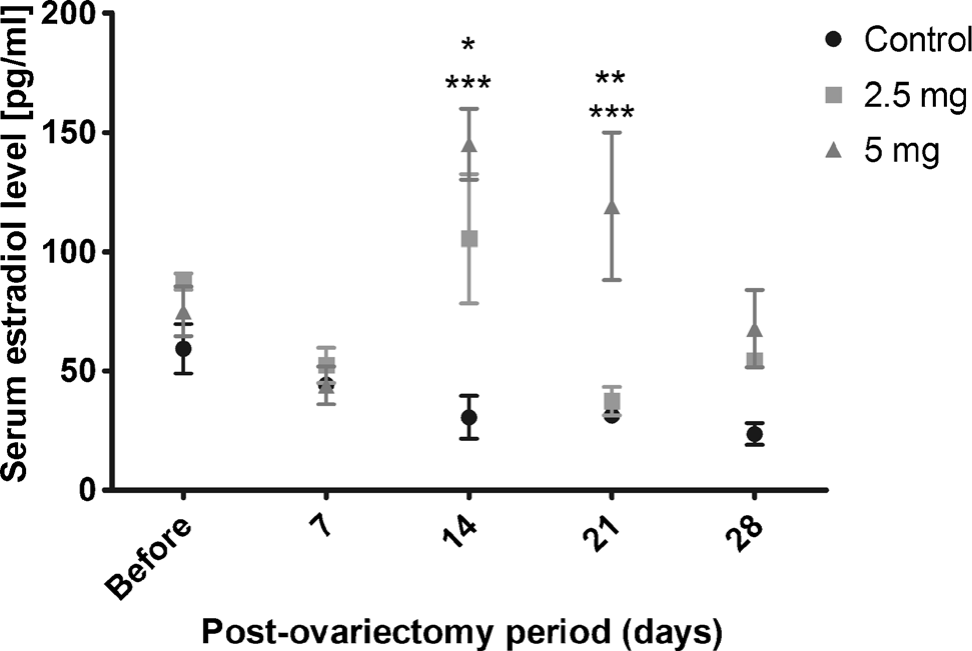
Measurements of serum E2 concentrations. Two-way ANOVA with the Bonferroni post hoc test was applied for comparison between the non-implanted control group (*n* = 4), ovariectomized group, and animals with 2.5 mg (*n* = 4) or 5 mg (*n* = 6) E2 rod-treated: ***-E2 rod-implanted 5 mg group vs. control (*p* < 0.001), *-E2 rod-implanted 2.5 mg group vs. control (*p* < 0.05), **-E2- rod- implanted group 2.5 mg vs. E2 rod-implanted group 5 mg (*p* < 0.01).

The E2 concentration in serum was measured for 4 weeks as effectively drawing blood from the ophthalmic artery was not possible. The formation of a scar was observed in the puncture site (Fig. 10).

In each period after ovariectomy the E2 level in serum was statistically significant for animals with implanted rods with E2. The highest increase in the E2 concentration was noted 14 days after implantation of each kind of rod, i.e. rods with doses of 2.5 and 5 mg. Rods with a dose of 5 mg caused a greater increase of the E2 concentration than rods with a dose of 2.5 mg. Moreover, it should be pointed out that the E2 levels were highly diversified in individual animals after rod implantation in the same time period (Fig. 10).

#### Tissue Morphology

**Fig. 11:**
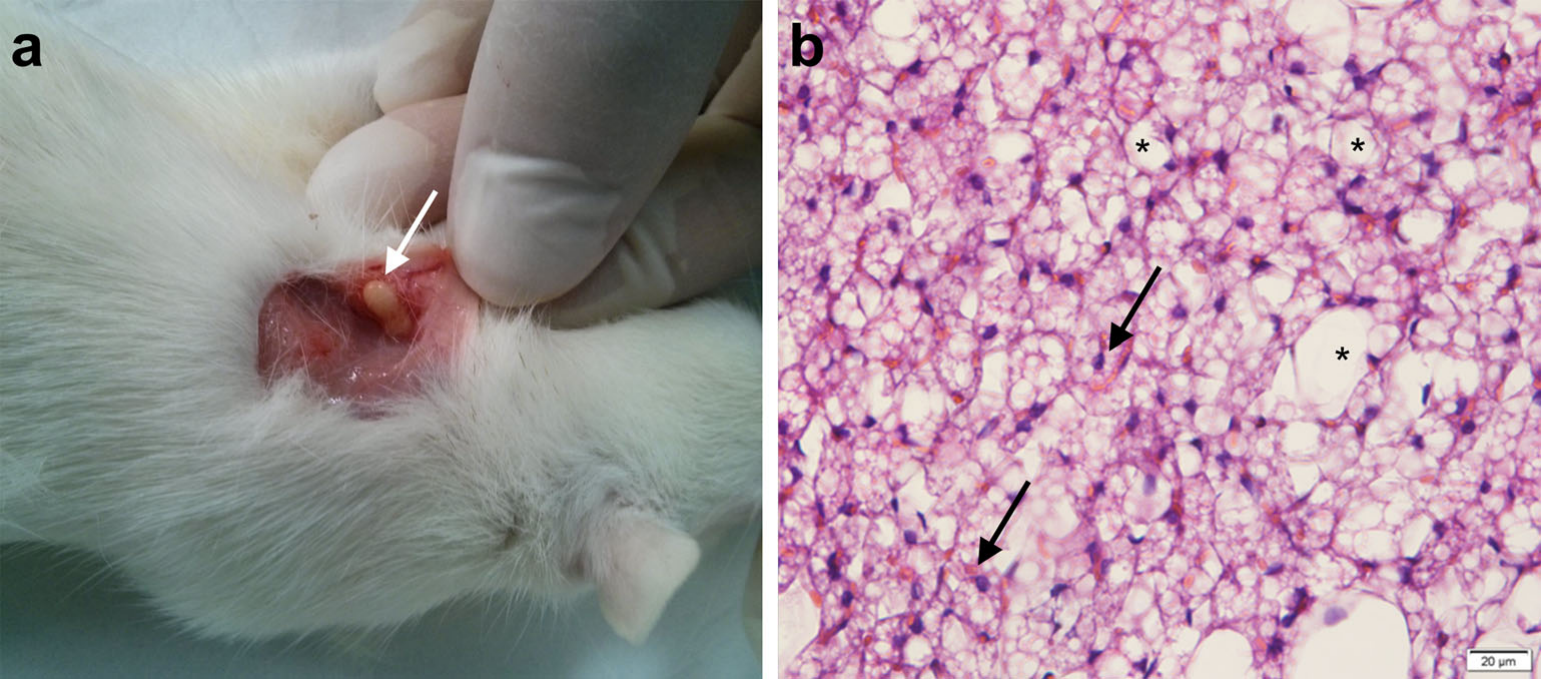
A view of rod explantation (**a** – *white arrow*) and histopathology of the tissue from the place of the explanted rod (magnification × 40) (**b**). *Black arrows* - fibroblast nucleus; *black stars* – adipocytes.

A microscopic study from the place of explantation revealed connective tissue with a visible fibroblast nucleus and adipocytes. No inflammation, necrosis and exudate were visible in tissue adjacent to the site of the rod implantation (Fig. 11a). Microscopic analysis revealed no signs of inflammation in the tissue. White blood cells and necrosis were not observed in any of the samples (Fig. 11b).

## Discussion

In this paper a novel conception has been developed in drug technology based on a terpolymer with shape-memory synthesized with the use of a low-toxic initiator. This formulation may guarantee lower invasiveness, regular release and lower irritation of tissues and may thus be suitable as a future treatment strategy for neurological disorders.

Designing the pharmaceutical formulation is the most important stage in the development of novel formulations. Thermoplastic polymers can be processed in various ways. One of these is the extrusion method. Solid formulations should be preferred for prolonged release so that explantation is possible in emergency cases. A rod is one of these applied formulations. It may be administered by needle from a pre-filled syringe or by an implanter.

It should be pointed out that there is a high risk of thermal degradation of the drug substance and polymer carrier during this extrusion process. Therefore, in this paper thermal analysis was performed for E2 and P(L-LA:GA:TMC) to determine the processing conditions.

It was noticed that the extrusion process of P(L-LA:GA:TMC) rods with E2 may be carried out at a temperature of 105°C. Maintaining stable conditions at this value is important for E2. Hydrogen-bonded water and lattice water in E2 will be maintained and changes between polymorphic crystal forms of E2 will not occur (Fig. 1) (30,31). Therefore, proposed conditions proved to be suitable.

Additionally, these conditions will not significantly change the terpolymer properties. The DSC curves of P(L-LA:GA:TMC) raw powder did not revealed any significant thermal event, which indicated an amorphous character of the applied terpolymer (Fig. 1). Moreover, it should be pointed out that the use of crystalline drug substance create a non-homogeneous formulation with amorphous polymer. Crystalline character was revealed for E2 and non-homogenuous structure of rods with E2 before degradation was showed in the observations performed by optical microscopy of the rods (Fig. 3).

The effectiveness of pharmacological interventions in the treatment of neurological disorders requires regular drug administration, thus the application of an implantable drug delivery system has many advantages over oral therapy. It has been stated that aliphatic polyesters such as polylactic acid, PLGA, and poly-ε-caprolactone are most often recommended in hormonal therapy with E2 (8,13–16). The release ratio depends on the degradation mechanism. It is commonly known that degradation of pointed aliphatic polyesters is based on bulk erosion through hydrolysis of the ester bonds. For most substances, sigmoidal release is observed during bulk erosion, however, it also depends on substance features (32).

In this paper, an almost linear E2 release with the burst effect from P(L-LA-GA:TMC) was observed (Fig. 4). However, this phenomenon did not influence release character. Zero-order release mainly results, which is preferred feature from therapeutic point of view.

An analogous mechanism of E2 release, i.e. the zero-order pattern, from the PLGA (50:50) carriers was revealed by another study. Optimal release was revealed for polymers with a molecular weight of 45.0 kDa. In that study the terpolymer possessed a similar value of this parameter. Moreover, the authors claimed that the release rate of E2 was dependent on the molecular weight of the polymer carrier and its chain composition. They proved that an increase in *M*_*n*_ in the range of 14.5–213 kDa of the PLGA carriers caused a significant decrease in the release rate of E2 (33). It should be pointed out that the P(L-LA:GA:TMC) applied here possessed *M*_*n*_ with a similar value, i.e. 59 kDA. A similar releasing profile was also presented in other studies (34,35).

In this paper, terpolymer P(L-LA:GA:TMC) was applied. In the case of LA and GA in copolymers, degradation takes place in the bulk. However, the degradation of aliphatic P(TMC) undergoes surface erosion. Therefore, the release process is controlled both by the structure and surface. It may influence the release mechanism controlled more by the surface than in the case of the PLGA copolymer.

Furthermore, it should be emphasized that zero-order release results from some given feature. The first is a lack of interaction between substance and polymer, which was indicated in this study. A comparison of the E2 - P(L-LA:GA:TMC) infrared spectrum with the algebraic sum of P(L-LA:GA:TMC) and E2 spectra showed no significant differences in all regions of the spectra (Fig. 2). It may thus be stated that the form of the composite does not favor linear release. However, a key role is played by the lack of interactions of the drug substance with the polymer.

There is no doubt that the amorphous character of the applied terpolymer will favor this release model. In the first heating run no endothermal events were noted. However, the loading of E2 into P(L-LA:GA:TMC) influenced the appearance of an endotherm, which may resulted from the presence of drug substance. It may be confirm by gradual disappearance of this endotherm during degradation. However, novel endotherms were noted, which may point to the crystallization process of the amorphous terpolymer. Generally, a stable and steady degradation was observed, which may influence linear release. However, derogations in release profile were also visible. Changes in the content of monomeric units in the terpolymer were noted. Advanced degradation was observed of glycolidyl units that were more hydrophilic than the lactidyl units. Moreover, only a meaningful steady decrease of *l*_*LL*_ was noted during the whole process of degradation (Table I). This phenomenon may influence the derogations in the linear character of the release profile. In the case of the other parameters, a gradual decrease in *T*_*g*_ and *M*_*n*_ *,* and a gradual increase in the values of *WU* and *WL* were also observed during the degradation process. This may also favor a linear release of E2 (Table I). The listed changes are typical of the degradation processes of aliphatic polyesters (18,36). However, the observed derogations in release profile of E2 between 57 and 92 days might result from significant changes in *T*_*g*_, *M*_*n*_, *WL* and *WU* in this period (Table I).

The morphological study performed by SEM did not reveal any unfavorable features, i.e. an evident porous surface or slits and cracks which might predispose uncontrolled release (Fig. 9).

The proposed solution of the novel E2 formulation indicated that the P(L-LA:GA:TMC) rods with E2 significantly increased its level in serum after ovariectomy (Fig. 9). However, the E2 concentration in serum in individual animals was highly diversified in the same period group after implantation, which shows that the animals reacted to the therapy in various ways. However, it should be emphasized that E2 was still secreted by the adrenal cortex after ovariectomy. Therefore, the observed E2 concentration was a total level, i.e. secreted endogenously and administered by the rods. Furthermore, no inflammation reactions, necrosis and exudate in tissue adjacent to the site of rod implantation were revealed (Fig. 11), which confirmed the low toxicity of the zirconium complexes.

## Conclusion

In conclusion, both the *in vitro* and *in vivo* study showed that shape-memory P(L-LA:GA:TMC) rods with E2 are an adequate proposal for further research in the field of neurological disorders and therapy. A stable and steady degradation process ensured linear release of E2 from the rods. This guaranteed prolonged release independently of its concentration. Rods may guarantee low invasiveness, regular release and low irritation of tissue. Moreover, the application of zirconium complexes resulted in no adverse effects caused by the polymer. Usage of the extrusion method was appropriate in order to obtain rods which allowed us to successfully apply them in animals, thus at the same time indicating their therapeutic applicability.

## Abbreviations

*∆H*: Enthalpy
*D*: Molecular weight distribution
DSC: Differential scanning calorimetry
E2: 17-β-estradiol
*F*_*GG*_: Molar percentage of glycolidyl units
*F*_*LL*_: Molar percentage of lactidyl units
FTIR: Fourier transform infrared spectroscopy
*F*_*TMC*_: Molar percentage of carbonate units
GPC: Gel permeation chromatography
k_0_: Zero-order release constant (release rate)
*l*_*GG*_: Average length of glycolidyl blocks
*l*_*LL*_: Average length of lactide blocks
*l*_*TMC*_: Average length of carbonate blocks
*M*_*n*_: Molecular weight
NMR: Nuclear magnetic resonance spectroscopy
P(L-LA:GA:TMC): Poly(L-lactide-co-glycolide-trimethylenecarbonate)
PBS: Phosphate buffered saline
PLGA: Poly(lactide-co-glycolide)
Q_0_: Initial amount released in time t = 0
Q_t_: Cumulative amount released in time t
SEM: Scanning electron microscopy
*T*_*g*_: Glass transitions temperature
*T*_*m*_: Melting temperature
*WL*: Weight loss
*WU*: Water uptake
Zr(Acac)_4_: Zirconium (IV) acetylacetonate
